# Porcine Sialoadhesin (CD169/Siglec-1) Is an Endocytic Receptor that Allows Targeted Delivery of Toxins and Antigens to Macrophages

**DOI:** 10.1371/journal.pone.0016827

**Published:** 2011-02-16

**Authors:** Peter L. Delputte, Hanne Van Gorp, Herman W. Favoreel, Inge Hoebeke, Iris Delrue, Hannah Dewerchin, Frank Verdonck, Bruno Verhasselt, Eric Cox, Hans J. Nauwynck

**Affiliations:** 1 Laboratory of Virology, Department of Virology, Parasitology, and Immunology, Faculty of Veterinary Medicine, Ghent University, Merelbeke, Belgium; 2 Laboratory of Immunology, Department of Virology, Parasitology, and Immunology, Faculty of Veterinary Medicine, Ghent University, Merelbeke, Belgium; 3 Department of Clinical Chemistry, Microbiology and Immunology, Faculty of Medicine and Health Science, Ghent University, Ghent, Belgium; University of Nebraska Medical Center, United States of America

## Abstract

Sialoadhesin is exclusively expressed on specific subpopulations of macrophages. Since sialoadhesin-positive macrophages are involved in inflammatory autoimmune diseases, such as multiple sclerosis, and potentially in the generation of immune responses, targeted delivery of drugs, toxins or antigens via sialoadhesin-specific immunoconjugates may prove a useful therapeutic strategy. Originally, sialoadhesin was characterized as a lymphocyte adhesion molecule, though recently its involvement in internalization of sialic acid carrying pathogens was shown, suggesting that sialoadhesin is an endocytic receptor. In this report, we show that porcine sialoadhesin-specific antibodies and F(ab')_2_ fragments trigger sialoadhesin internalization, both in primary porcine macrophages and in cells expressing recombinant porcine sialoadhesin. Using chemical inhibitors, double immunofluorescence stainings and dominant-negative constructs, porcine sialoadhesin internalization was shown to be clathrin- and Eps15-dependent and to result in targeting to early endosomes but not lysosomes. Besides characterizing the sialoadhesin endocytosis mechanism, two sialoadhesin-specific immunoconjugates were evaluated. We observed that porcine sialoadhesin-specific immunotoxins efficiently kill sialoadhesin-expressing macrophages. Furthermore, porcine sialoadhesin-specific albumin immunoconjugates were shown to be internalized in macrophages and immunization with these immunoconjugates resulted in a rapid and robust induction of albumin-specific antibodies, this compared to immunization with albumin alone. Together, these data expand sialoadhesin functionality and show that it can function as an endocytic receptor, a feature that cannot only be misused by sialic acid carrying pathogens, but that may also be used for specific targeting of toxins or antigens to sialoadhesin-expressing macrophages.

## Introduction

Sialoadhesin (Siglec-1, CD169, or Sn) was initially identified as a sialic acid-dependent sheep erythrocyte receptor (SER) on resident bone marrow cells of mice, and is now also characterized in man, rat and swine [1]–[5]. Sn belongs to the family of sialic acid binding immunoglobulin-like lectins (siglecs) which are expressed, with exclusion of MAG (Siglec-4), on distinct subsets of haematopoietic cells [6]. Sn is expressed only on specific subsets of tissue macrophages that are found mostly in spleen, lymph nodes, bone marrow, liver, colon and lungs [3], [5], [7]–[9]. High Sn expression has also been detected on inflammatory macrophages in tissues from patients with rheumatoid arthritis, and on infiltrating macrophages that make close contact with breast carcinoma cells, suggesting a role for Sn or Sn-positive macrophages in these diseases [3],[10]. Recently, Sn deficient mice have been generated and their use in murine models of inflammatory autoimmune diseases, such as multiple sclerosis [11], further supports the concept that Sn-positive macrophages may play a role in regulation of immune responses [12].

Almost all siglecs have one or more cytosolic tyrosine-based motifs that are implicated in signal transduction and/or endocytosis [13]. Intriguingly, Sn lacks obvious tyrosine-based motifs, nevertheless recent data provide evidence for a role of Sn in receptor-mediated internalization processes and show that pathogens that carry sialic acids can be internalized into Sn-expressing macrophages. Indeed, porcine Sn (pSn) is involved in attachment and internalization of the porcine arterivirus [5], [14]–[17]. Further, it was shown that alveolar macrophages that express pSn internalize a Sn-specific monoclonal antibody (mAb) [5]. Mouse macrophages expressing murine Sn (mSn), and cells expressing recombinant mSn were also shown to be involved in binding and phagocytosis of sialylated *Neisseria meningitides* [18]. Although initially characterized as a non-phagocytic adhesion molecule involved in cell-cell interactions [8], [19], [20], these data indicate the involvement of Sn in internalization processes, which may have implications for the understanding of its physiological role.

The possible role of Sn in an internalization process and its restricted expression pattern on macrophages implicate potential use of this protein in specific macrophage targeting of antigens, toxins, drugs or other molecules, either to specifically eliminate, activate or deactivate macrophages. Seen the potential of this newly attributed property of Sn, this study aimed to characterize the endocytic properties of pSn upon binding of Sn-specific antibodies and to analyze the potential of this receptor as a macrophage-specific molecule allowing targeting of toxins and antigens.

## Results

### Confocal microscopical analysis of antibody-induced Sn internalization in primary porcine macrophages and cells expressing recombinant pSn

**Figure 1:**
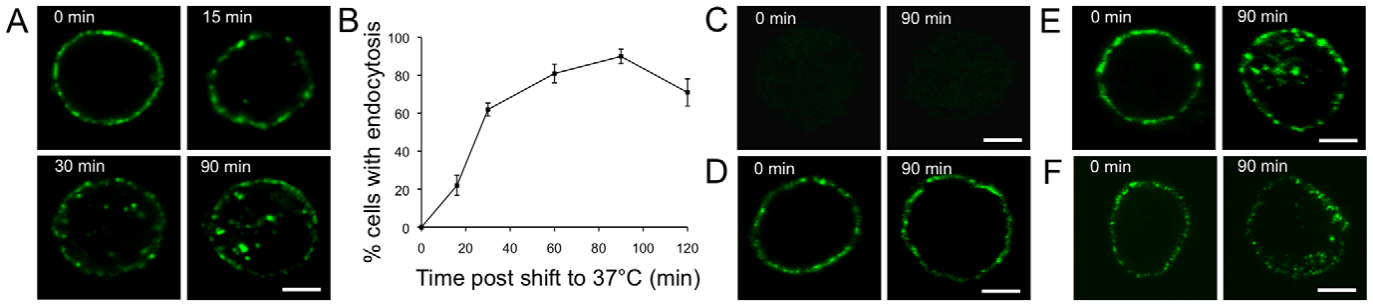
Kinetics of antibody-induced pSn internalization. (A) Confocal microscopical analysis of mAb 41D3-induced Sn internalization in primary porcine macrophages. Cells were incubated for the indicated times with mAb 41D3 at 37°C, fixed, permeabilized and stained with FITC-labelled goat-anti-mouse IgG. Images represent a single confocal z-section through the middle of the cell. (B) Kinetics of the percentage of macrophages with internalized Sn at different time points after incubation of macrophages at 37°C with mAb 41D3. Data represent the means ± standard deviations of 3 independent experiments. (C) Isotype matched control mAb 13D12 (gD of pseudorabies virus) shows no staining of primary macrophages. (D) Antibody internalization is not observed in primary macrophages stimulated with control mAb 74-22-15 (SWC3). (E) 41D3 F(ab')_2_ fragments lacking the Fc domain are internalized in primary macrophages. (F) Sn-specific mAb 41D3 induces internalization in CHO cells expressing recombinant pSn. Scale bar: 5 µm.

To study Sn endocytosis, porcine macrophages were incubated with the Sn-specific mAb 41D3 and at different time points cells were fixed and stained. At time 0, a clear membrane staining was observed, and none of the macrophages contained Sn-positive vesicles in the cytoplasm (Fig. 1a–b). With increasing time, the number of cells which internalized Sn increased to reach a maximum of 90% at 90 min (Fig. 1a–b). At early time points, endocytic vesicles were mainly present in the vicinity of the plasma membrane, while with increasing time, endocytosed Sn was localized closer to the perinuclear region (Fig. 1a). As a control, macrophages were incubated with irrelevant, isotype matched mAb 13D12 (gD of pseudorabies virus), or mAb 74-22-15 (SWC3 on macrophages). Cells incubated with mAb 13D12 showed no staining (Fig. 1c), while mAb 74-22-15 incubated cells showed exclusive plasma membrane staining at all time points examined (Fig. 1d). To exclude the potential involvement of Fc receptors in 41D3-induced internalization, macrophages were incubated with 41D3 F(ab')_2_ fragments, showing clear internalization (Fig. 1e and Fig. S1). In addition, 41D3 was added to CHO-Sn cells expressing recombinant pSn, but lacking Fc receptors. Again 41D3 was internalized, confirming that Fc receptors are not required for 41D3-induced internalization (Fig. 1f).

### Flow-cytometric analysis of pSn endocytosis

**Figure 2:**
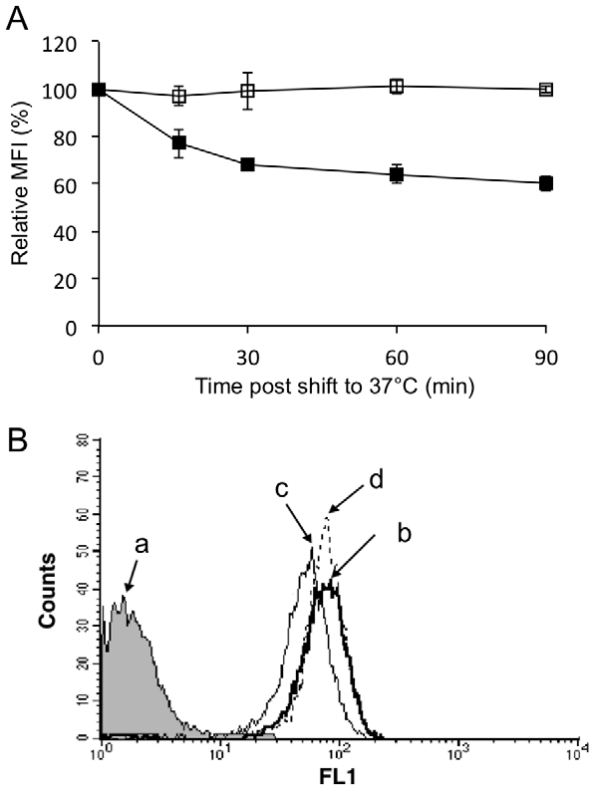
Flow cytometric analysis of pSn internalization. (A) Cell surface expression of Sn was quantified at different time points after incubation at 37°C with either the Sn-specific antibody 41D3 or the irrelevant, isotype matched control antibody 13D12. The level of surface expression is expressed as MFI, with time 0 as reference point. pSn internalization was assessed via incubation with the pSn-specific mAb 41D3 (black squares), followed by the staining consisting of a FITC-labelled secondary antibody. As a control, cells were incubated with the control antibody 13D12 (open squares), followed by the staining consisting of 41D3 as primary antibody and a FITC-labelled secondary antibody. Staining was performed at the different time points as indicated. Data represent the means ± standard deviations of 3 independent experiments. (B) Representative histograms of analysis of Sn internalization in porcine macrophages. (a) Staining of macrophages with isotype matched (IgG_1_), irrelevant control mAb. To analyze antibody-induced Sn internalization, macrophages were incubated for 1 h with mAb 41D3 at (b) 4°C or at (c) 37°C, followed by staining surface bound antibodies (no permeabilization) with FITC-labelled goat-anti-mouse IgG to detect non-internalized mAb 41D3 on the cell surface. (d) To confirm that antibodies were internalized into the macrophage, cells were incubated for 1 h with mAb 41D3 at 37°C, followed by fixation, permeabilization and staining with FITC-labelled goat-anti-mouse IgG to detect both internalized and cell surface bound mAb 41D3 (FL1 – logarithmic scale).

Antibody-induced pSn endocytosis was only partial, since confocal microscopical analysis showed that, together with visible internalized Sn, a clear plasma membrane staining could still be observed, even at 90 min when endocytosis was visible in most cells (Fig. 1b). To estimate the amount of internalized Sn, flow cytometry was used. With increasing time, a clear reduction in the mean fluorescence intensity (MFI) of surface Sn could be observed (Fig. 2a, black squares), with a maximum reduction of surface fluorescence at 90 min. When cells were permeabilized prior to staining, so that both surface bound and internalized Sn were stained, the MFI was identical at 0 and 90 min, indicating that the observed reduction in fluorescence is not due to shedding of antibody-antigen complexes (Fig. 2b). Macrophages stimulated with mAb 13D12 showed no reduction in surface Sn staining (Fig. 2a, open squares).

### Mechanism of antibody-induced pSn internalization in primary macrophages

**Figure 3:**
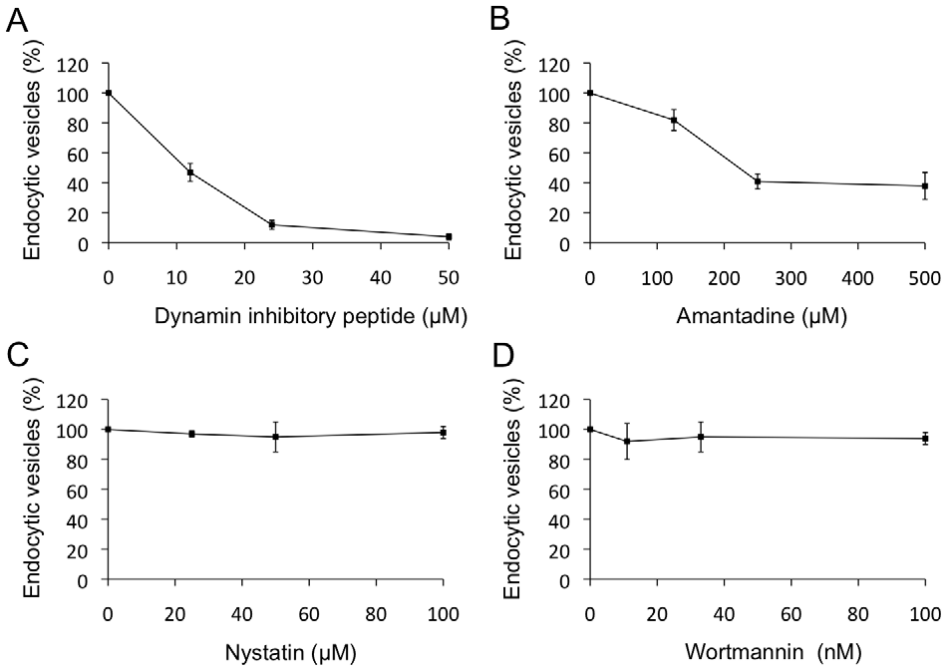
Effect of different inhibitors on antibody-induced pSn endocytosis. Macrophages were stimulated with mAb 41D3 in the presence of different concentrations of (A) dynamin inhibitory peptide which blocks clathrin- and lipid raft/caveolae-dependent endocytosis and phagocytosis, (B) amantadine, which interferes with clathrin-mediated endocytosis, (C) nystatin, which blocks lipid raft/caveolae-mediated endocytosis, and (D) wortmannin, a blocker of phagocytosis. At 60 min post internalization, cells were fixed and stained. The number of internalized vesicles was quantified and expressed as a percentage relative to the number of internalized vesicles in the absence of the inhibitor. Data represent the means ± standard deviations of 3 independent experiments.

Discrimination between the major endocytic pathways can be made on the basis of their differential sensitivity to pharmacological/chemical inhibitors [21], [22]. The mechanism of pSn endocytosis was therefore initially analyzed using inhibitors that block dynamin-dependent endocytosis (dynamin inhibitory peptide), clathrin-mediated endocytosis (amantadine), lipid raft/caveolae-mediated endocytosis (nystatin) and phagocytosis (wortmannin). The dynamin inhibitory peptide is an amphiphysin SH3 domain recombinant protein that competitively blocks binding of dynamin to amphiphysin, thereby preventing the recruitment of dynamin to clathrin-coated pits [23]–[25]. Using this peptide, a clear block in Sn internalization was observed, indicating that internalization occurs via a dynamin-dependent process (Fig. 3a). Also amantadine, which blocks the budding of clathrin-coated vesicles [26], [27], reduced Sn internalization in a dose-dependent way (Fig. 3b). In contrast, nystatin and wortmannin had no effect (Fig. 3c–d), with nystatin sequestering cholesterol from microdomains and accumulating it in aggregates, and wortmannin inhibiting phosphatidylinositol 3-kinases (PI3Ks) respectively [21]. The absence of an effect of nystatin is in agreement with the observation that pSn is not localized to cholesterol enriched lipid raft microdomains (see Results S1 and Fig. S2). Together, these data suggest that pSn endocytosis is mediated via clathrin-coated vesicles.

**Figure 4:**
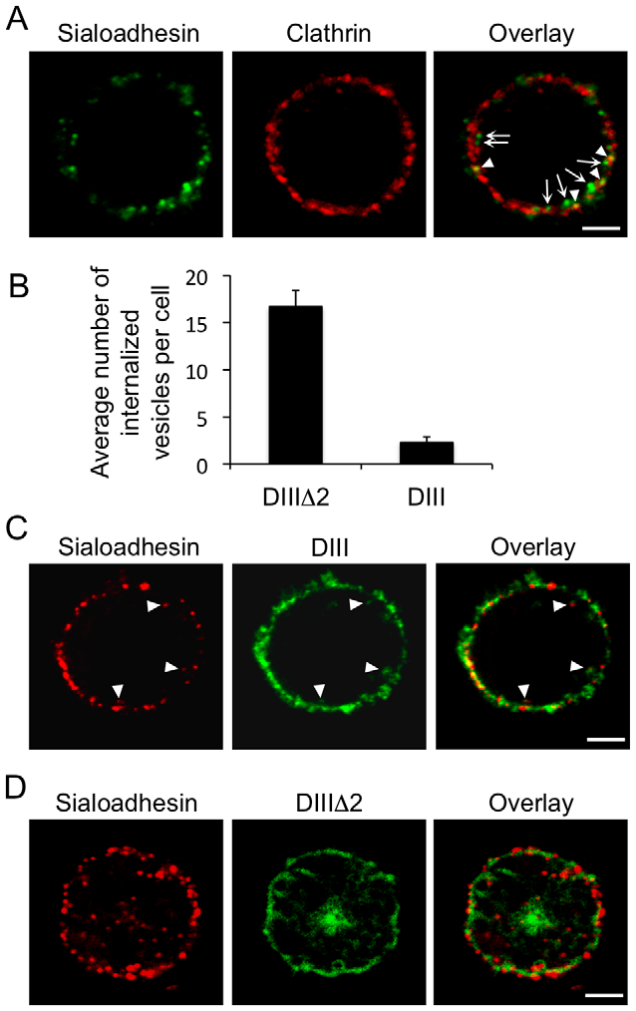
Antibody-induced pSn endocytosis is clathrin-mediated. (A) Sn endocytosis was stimulated by incubating porcine macrophages with mAb 41D3 at 37°C. Cells were fixed 15 min after the stimulation and stained for both Sn (green) and clathrin (red). (B) Effect of lentiviral transduction of primary porcine macrophages with dominant-negative Eps15 (Eps15-DIII) and the control Eps15-DIIIΔ2 on Sn internalization in macrophages. (C–D) Representative confocal images of Sn internalization in macrophages expressing dominant-negative Eps15 (DIII) or inactive Eps15 (DIIIΔ2). Arrowheads indicate Sn internalization in cells expressing Eps15DIII, which co-localizes with Eps15 as shown by the yellow colour in the overlay. Scale bar: 5 µm.

Since the results with the inhibitors indicate that pSn is internalized via clathrin-mediated endocytosis, double immunofluorescence stainings for both Sn and clathrin were performed to confirm the potential involvement of clathrin. A clear co-localization between Sn and clathrin could be observed during Sn invagination from the plasma membrane, further suggesting that Sn internalization is clathrin-mediated (Fig. 4a, arrowheads). Vesicles that were completely internalized in the cytoplasm no longer co-localized with clathrin, indicating that they released their clathrin coat (Fig. 4a, arrows). Finally, a dominant-negative form of Eps15 (Eps15-DIII) that inhibits clathrin-mediated endocytosis was transduced into Sn-expressing macrophages using a lentiviral transduction system. As a control, a non-functional Eps15 construct (Eps15-DIIIΔ2) was used. Eps15-DIII transduced cells showed a 90% reduction in internalization compared to Eps15-DIIIΔ2 transduced cells (Fig. 4b–d), confirming the importance of Eps15 and clathrin for antibody-induced Sn internalization in porcine macrophages.

### Intracellular movement of pSn is dependent on dynein and targets early endosomes

**Figure 5:**
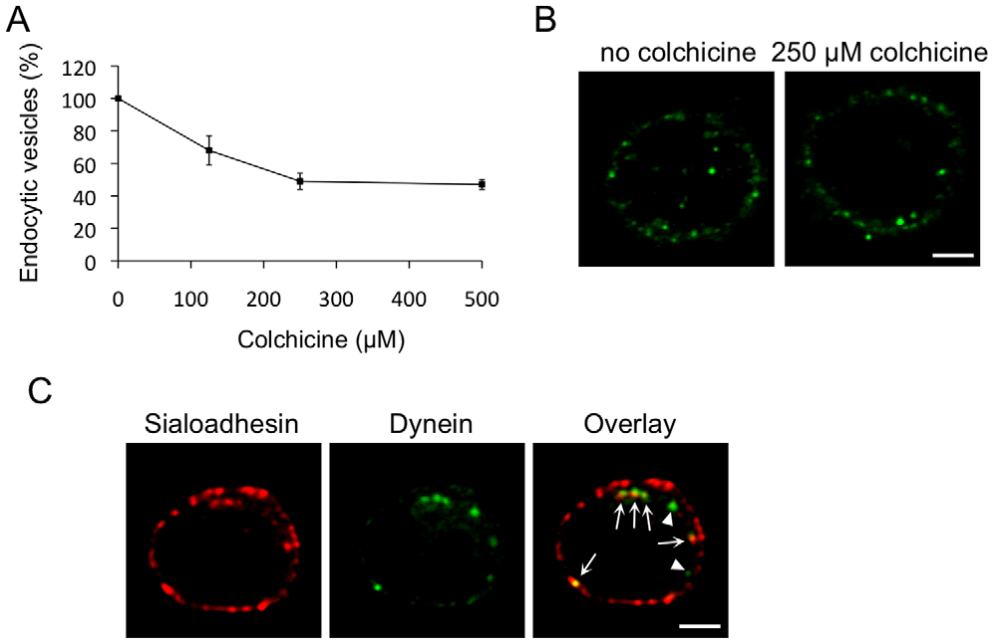
Microtubules and the motor protein dynein are involved in intracellular transport of Sn-positive vesicles. (A) Macrophages were stimulated with mAb 41D3 in the presence of different concentrations of the microtubule disrupting agent colchicine. The number of internalized vesicles was quantified and expressed as a percentage relative to the number of internalized vesicles in the absence of the inhibitor. Data represent the means ± standard deviations of 3 independent experiments. (B) Confocal analysis of mAb 41D3 stimulated pSn endocytosis in the presence or absence of colchicine. Colchicine treatment clearly resulted in a block of the intracellular Sn movement, and Sn-positive vesicles remained close to the plasma membrane. (C) Confocal analysis of a double immunofluorescence staining of Sn (red) and dynein (green) in mAb 41D3 stimulated macrophages. Partial co-localization is observed between Sn-positive vesicles and dynein, as shown by the arrows in the overlay. Arrowheads indicate internalized Sn that does not co-localize with dynein. Images represent a single confocal z-section through the middle of the cell. Scale bar: 5 µm.

The possible role of microtubules and dynein, a motor protein involved in movement of vesicles along microtubules, in the intracellular transport of internalized Sn was analyzed by treating cells with colchicine, a microtubule disrupting agent, and via a double immunofluorescence staining for Sn and dynein. Colchicine treatment of porcine macrophages still allowed Sn internalization, although at lower levels than in the absence of colchicine (Fig. 5a–b). However, when colchicine was added, Sn remained close to the plasma membrane and did not move in the direction of the perinuclear region (Fig. 5a–b). Immunofluorescence stainings for both Sn and dynein showed that Sn-positive vesicles were in close contact with dynein, since the dynein fluorescence signal partially overlapped with the Sn fluorescence signal, as indicated by the yellow colour in the overlay (Fig. 5c), suggesting that dynein mediates intracellular transport of internalized pSn along microtubules.

**Figure 6:**
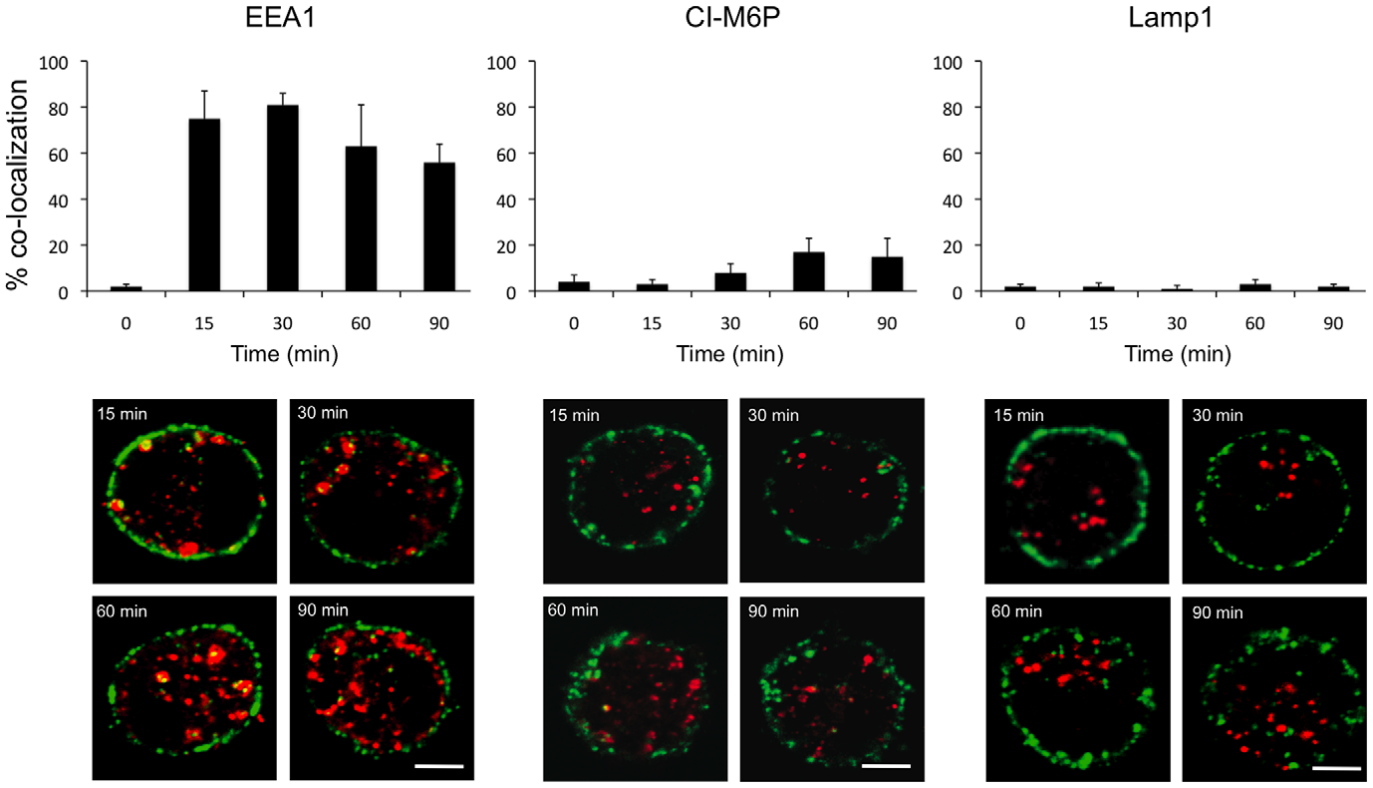
Analysis of co-localization between internalized pSn and early endosomes (EEA1), late endosomes (CI-M6P) or lysosomes (Lamp1) in macrophages. Co-localization between internalized pSn (green) and EEA1, CI-M6P or Lamp1 (red) was calculated from confocal z-sections of 25 randomly selected cells at the indicated time points. Data represent the means ± standard deviations of 3 independent experiments. Representative images of macrophages at different time points during internalization are shown as overlays of the red and green signal with a yellow colour indicating co-localization. Scale bar, 5 µm.

To analyze the intracellular localization of internalized, Sn-specific antibodies, double immunofluorescence stainings were performed for mAb 41D3 and EEA1, CI-M6P or Lamp1, markers for early endosomes, late endosomes and lysosomes respectively. At 15 and 30 min after the start of internalization, the majority of internalized antibodies (>80%) were localized to early endosomes (Fig. 6). Starting from 60 min, co-localization with early endosomes diminished to approximately 60%, while from this point co-localization was observed with late endosomes with a maximum of 20%. No co-localization of internalized antibodies with lysosomes was detected at any time tested. From these experiments it is concluded that pSn internalization mainly targets to early endosomes and that internalized ligands reside for prolonged times in this compartment.

### Porcine Sn-specific immunotoxins kill macrophages

**Figure 7:**
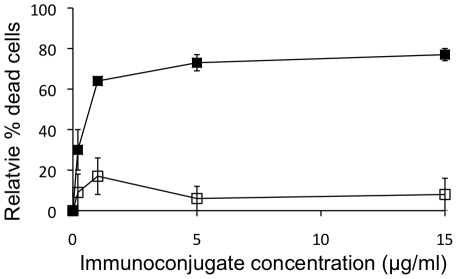
Effect of immunotoxins on macrophage viability. Macrophages were incubated with different concentrations of Sn-specific (saporin linked to mAb 41D3; black squares) or control immunotoxins (isotype control mAb linked to saporin; open squares). After 10 hrs of treatment, the relative percentages of dead cells were determined, with concentration 0 as reference point.

To evaluate the potential to use Sn endocytosis to specifically target molecules to macrophages, immunotoxins were constructed consisting of the pSn-specific mAb 41D3 and the ribosome inactivating protein saporin. On its own, saporin is not able to enter cells, but it can be co-internalized following conjugation with antibodies that recognize cell surface proteins [28]. The disulfide bond introduced to link antibody and saporin allows dissociation of the toxin upon internalization, which is essential for its activity. Saporin conjugated to pSn-specific mAb 41D3 could efficiently kill macrophages in a dose-dependent manner. At a concentration of 1 µg/ml immunotoxin, more than 60% of the cells were dead after 10 hrs of incubation, while almost 80% of the cells were killed using 15 µg/ml immunotoxin (Fig. 7). Control immunoconjugates (irrelevant, isotype matched antibody) had no significant effect on cell viability, even at the highest concentrations used.

### Porcine Sn-specific HSA immunoconjugates are targeted to Sn-expressing cells, which results in enhanced antibody responses upon immunization

**Figure 8:**
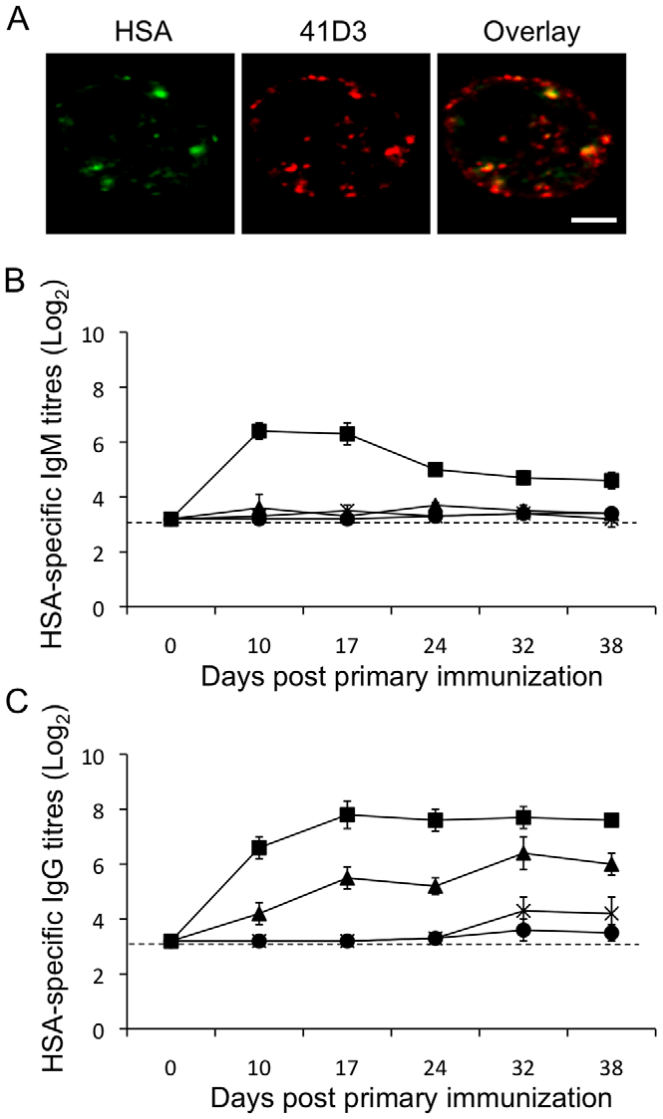
Targeting of the HSA antigen to Sn-positive porcine macrophages. (A) Confocal microscopical analysis of primary macrophages incubated for 60 min at 37°C with mAb 41D3-HSA immunoconjugates. HSA was detected with biotinylated HSA-specific pig serum and FITC-labelled streptavidin (green) and mAb 41D3 was stained with TexasRed labelled goat-anti-mouse IgG (Red). Scale bar, 5 µm (B) and (C) Kinetics of HSA-specific IgM (B) and IgG (C) antibody titres upon immunization without adjuvant with Sn-specific immunoconjugates (mAb 41D3-HSA; squares), with control immunoconjugates (control mAb 13D12-HSA; triangles), with HSA and 41D3 not conjugated (crosses) or HSA alone (circles). Data are means ± SEM of 6 (41D3-HSA) or 3 animals (control mAb-HSA, HSA and 41D3 not conjugated, and HSA alone). Dashed line represents the detection limit.

Antibody-targeted vaccines are used to deliver an antigen to professional antigen-presenting cells to induce or enhance cellular and/or humoral immunity to the antigen [29], [30]. Sn-positive cells can be found in the spleen and lymph nodes where they can trap blood and lymph-born antigens respectively, with the capacity to induce T-cell immunity [31], [32]. Given its interesting expression profile and capacity to internalize antibodies and antibody-conjugates, we further analyzed if this property could be used to specifically target antigens to Sn-positive cells thereby inducing humoral immunity. Therefore, the antigen human serum albumin (HSA) was chemically cross-linked via a thio-ether bond to the Sn-specific mAb 41D3, or to the irrelevant, isotype matched (IgG_1_) control mAb 13D12. Incubation of primary, Sn-expressing porcine macrophages with the Sn-specific immunoconjugate resulted in uptake of HSA inside the macrophage (Fig. 8a). In addition, a clear co-localization was seen between internalized Sn-specific antibodies and HSA, confirming that HSA internalization was mediated by the Sn-specific mAb. In contrast, HSA coupled to the control mAb was not internalized in macrophages, or only in very low amounts in some cells. To investigate whether specific targeting of HSA immunoconjugates in vivo could have an effect on HSA-specific immune responses, pigs were immunized with HSA linked to Sn-specific mAb 41D3, HSA linked to a control mAb, HSA and mAb 41D3 not conjugated or with HSA alone. Half of the sample was injected intravenously and the other half intramuscularly to target Sn-positive cells in the spleen and in the lymph nodes respectively. Analysis of HSA-specific antibodies in serum at different time points post immunization revealed that animals immunized with the Sn-specific immunoconjugate had the highest and fastest IgM and IgG immune responses during the course of the experiment (Fig. 8b–c), suggesting that specific targeting of antigens to Sn-expressing macrophages ameliorates antibody responses.

## Discussion

Previously, Sn was reported to be involved in the attachment and entry process of viruses [5], [14]–[17], [33]–[35], a bacterium [18], and potentially also a parasite [36]. Interestingly, all these pathogens carry sialic acid on their surface and the presence of this sugar is essential for their interaction with Sn. Although Sn was shown to be involved in internalization of these pathogens, these findings do not directly show beyond doubt the endocytic properties of Sn, since Sn might only mediate pathogen binding, while other receptors could engage internalization. Previously, pSn-specific antibodies were also shown to be internalized by primary macrophages, further indicating that Sn is a receptor with the capacity to internalize [5], [31]. So far, fundamental knowledge on the Sn-mediated entry process is lacking, therefore we aimed to investigate Sn in its function as an endocytic receptor and to explore its potential as a gateway for macrophage-directed therapeutics.

Upon addition of mAb 41D3, we observed that pSn together with the mAb was internalized into the cell. This internalization was clearly mediated by Sn and not by other macrophage receptors, since isotype matched control mAbs were not internalized in the cell and since 41D3 F(ab')_2_ fragments, lacking the Fc domain, are internalized similarly to intact 41D3. In addition, antibody-induced internalization was also observed in non-macrophage cell lines that express recombinant pSn, but that do not express Fc receptors. In the future, Fab fragments will be generated to assess whether receptor cross-linking is required for internalization, as well as the requirement of a ligand to trigger Sn internalization. Subsequently, the mechanism of 41D3-induced pSn internalization was explored. Using inhibitors, double immunofluorescence stainings, and lentiviral transduced dominant-negative Eps15 constructs, pSn endocytosis was shown to be clathrin-dependent. Clathrin-dependent endocytosis of cell surface receptors is known to depend on the presence of specific amino acid endocytosis motifs in the cytoplasmic domain of the internalized protein. Tyrosine- and di-leucine-based motifs are well-known for their interaction with adaptor proteins as a first step in endocytosis (reviewed by [37]). Such motifs however, are not present in the cytoplasmic domain of Sn. Interestingly, the absence of tyrosine-based internalization motifs makes Sn an exception in the family of siglecs, since most other siglecs identified to date were shown to contain tyrosine-based signalling motifs, and several of them, such as Siglec-2 (CD22) and Siglec-3 (CD33), have been shown to be internalization receptors [13], [38], [39]. Most recently, it has been published that apart from the well-known endocytic motifs, additional motifs await discovery [40], thus including the one driving Sn endocytosis.

There are major differences in the cytoplasmic domains of Sn of different species, both in amino acid sequence and length. So far, no potential, known clathrin-dependent internalization motifs were identified in the cytoplasmic domain of Sn of any species [3], [5], [8]. Shadee-Eestermans *et al*. [41] suggested the involvement of mSn in a receptor-mediated internalization process, since Sn could be detected in intracellular vesicles. In contrast however, antibody 3D6 directed against mSn is apparently not internalized in primary macrophages upon binding [42]. Clearly, a comparative analysis of antibody-induced internalization of Sn of other species and identification of internalization motifs involved, is needed to elucidate whether clathrin-dependent endocytosis is a general feature of Sn of different species, or whether this is a feature of pSn alone.

Confocal microscopical and flow cytometric analyses showed that Sn endocytosis appears as a rather slow process. After 30 min stimulation with mAb 41D3, approximately 20% of Sn was internalized, and a maximum of 41% was internalized at 90 min. Why not all Sn is internalized upon mAb 41D3 stimulation is not known. One possible explanation why only a fraction is internalized is that two or more forms of pSn may be present in the plasma membrane of macrophages, each being recognized by mAb 41D3, but having different internalization capacities. Such putative different forms may result from alternative splicing. Alternative splicing of Sn transcripts was previously described for mSn, but these transcripts were shown to encode for soluble, secreted variants of Sn, and are thus unlikely to be involved in Sn internalization from the membrane [8]. If different isoforms of Sn with different endocytosis capacity exist, the difference may very well be located in the cytoplasmic domain of the protein, since this domain may contain the information required for efficient internalization. Indeed, for the B-cell receptor Siglec-2 (CD22), which was shown to be internalized via clathrin-coated pits, alternative splicing was shown to result in two isoforms (CD22α and CD22β) with different cytoplasmic domains [43]–[45] and similarly, alternative splicing of Siglec-8 was shown to result in isoforms with different cytoplasmic domains [46]. The pSn gene has however not yet been characterized, it can thus not be excluded that alternative splicing could lead to expression of pSn isoforms with differences in the cytoplasmic domain, which possibly could explain the incomplete pSn internalization observed in our experiments.

In our study, inhibition of phagocytosis had no effect on monoclonal antibody-induced pSn internalization, which is in agreement with the characterization of mSn as a non-phagocytic receptor [2]. However, recent findings show the involvement of mSn in phagocytosis of sialylated *Neisseria meningitides* [18]. As suggested by the latter authors, mSn might act in synergy with another phagocytic receptor, or the experimental differences might be attributed to the size of the particle (erythrocytes or bacteria) that is presented to the Sn-expressing macrophages. Porcine Sn, here shown to mediate clathrin-dependent endocytosis, might thus also function as a phagocytic receptor under different experimental conditions, for example when larger particles coated with mAb 41D3 would be presented to the macrophages. Indeed, the involvement of a receptor in endocytosis does not prevent its functioning as a phagocytic receptor. Fc receptor internalization via phagocytosis or endocytosis was shown to be dependent on the size or multiplicity of the ligand presented to the cells [47], while the mannose receptor was shown to mediate either endocytosis or phagocytosis depending on the state of activation of the cell [48].

Not only Sn, but also other siglecs, such as Siglec-2 (CD22) and Siglec-3 (CD33) were shown to internalize specific monoclonal antibodies, with CD22 internalization being characterized as clathrin-mediated [39], [43], [44], [49], [50]. Interestingly, the capacity of CD22 to internalize specific monoclonal antibodies was shown to make this molecule an effective target for immunoconjugate therapy of B-cell malignancies [51]–[53]. Additionally, immunoconjugates targeting CD33 are currently a valuable option to treat patients with acute myeloid leukemia (AML) [54]. Recently, Siglec-H was also identified as an endocytic receptor that allows targeted delivery of antigens to murine plasmacytoid dendritic cell precursors, which results in the induction of CD8^+^ T-cells [55]. Similarly, a Sn-specific mAb was shown to efficiently induce T-cell proliferation compared to an aspecific control mAb, indicating the potential of pSn as a gateway molecule for antibody-targeted vaccines [31]. Together with our data, these promising results fuel the increasing interest in siglecs as targets for cell-directed immunotherapy as nicely exhibited in a recent review [13].

In this study, we showed that the capacity of pSn to mediate antibody-induced internalization can be used for specific targeting of toxins and antigens to macrophages. This targeted delivery of a cargo was shown to be functional, since Sn-specific immunotoxins allowed killing of macrophages. This property could potentially be of use in diseases were inflammatory, Sn-expressing macrophages might promote disease progression [3], [11], [56], [57]. Sn-specific immunotoxins would then have the potential to eliminate these cells, but further studies are needed to confirm these findings in vivo.

An interesting observation was the enhanced antibody response that was observed in animals immunized with immunoconjugates of the model antigen HSA linked to a Sn-specific mAb, compared to immunization with HSA alone or HSA linked to an irrelevant control mAb. For the Sn-specific conjugate, IgG antibody responses were faster and higher compared to the other constructs. Since the immunization was done in the absence of adjuvant, this could suggest that in vivo targeting to Sn might have adjuvant effects. This effect most likely results from the specific targeting to Sn-expressing cells, since immunization with both HSA and the Sn-specific mAb (not conjugated) resulted in a similar antibody response as immunization with HSA alone. Thus, Sn signalling induced by antibody-mediated cross-linking is not sufficient on its own to stimulate antigen-specific antibody responses. In contrast to HSA alone, the control HSA-conjugate did induce an IgG response, albeit less potent compared to the Sn-specific conjugate. This might be explained by Fc receptor-mediated effects or by the difference in size between the unconjugated and the conjugated HSA influencing the cellular uptake mechanism [29]. Conjugation of HSA to an antibody thus increases its immunogenicity, which is even further improved by targeting HSA to Sn. The specificity and mechanism responsible for this enhanced humoral immune response need further investigation, requiring in vivo insight in the cells responsible for antigen uptake, processing and presentation. In this context, it should be mentioned that although Sn is known as a macrophage-restricted surface molecule, rhinovirus infection seems to be able to induce Sn on dendritic cells (DCs) [58] and Sn seems to be expressed by some DCs in the lymph nodes [59]. Our finding that targeting to Sn-positive cells improves the humoral immune response emphasizes the importance of Sn-positive cells in immune responses. These cells appear to have multiple functions, since recently, Sn-positive marginal metallophilic macrophages in the spleen were shown to transfer antigens to cross-presenting dendritic cells resulting in cytotoxic T-cell immunity [60]. Also in the lymph nodes Sn-positive macrophages have been shown to trigger T-cell immunity [61]. Although the precise mechanisms involved remain to be elucidated, Sn seems a versatile and promising target to accomplish immuno-modulating therapy.

Our results, together with the findings of others [5], [18], [31], clearly expand Sn functionality and show that pSn not only functions as an adhesion receptor, but is also an endocytic receptor. This feature may be important for the, yet unknown, physiological function of this protein, but may also make it a gateway, not only for the porcine arterivirus, but also for other sialylated pathogens to gain entry in macrophages. Finally, our data also show that pSn can be used for targeted delivery of antigens or other molecules, which may result in an enhanced humoral immune response.

## Materials and Methods

### Ethics Statement

The experiments were authorized and supervised by the Ethical and Animal Welfare Committee of the Faculty of Veterinary Medicine of Ghent University. The named institution approved the experiments and provided a permit for this study (Permit Number: EC 2005/14).

### Cells, reagents and antibodies

Primary porcine alveolar macrophages were isolated from 4- to 6-week-old conventional Belgian Landrace pigs as described [62]. The cells were cultivated in RPMI-1640, supplemented with 10% foetal bovine serum (FBS), 2 mM L-glutamine (BDH Chemicals Ltd.), 1% non-essential amino acids (Gibco BRL), 1 mM sodium pyruvate (Gibco BRL) and antibiotics in a humidified 5% CO_2_ atmosphere at 37°C. For all experiments, macrophages were cultivated for 24 hrs before use. The CHO-Sn cells that stably express recombinant pSn were described earlier [15].

All products were purchased from Sigma, unless otherwise mentioned. The myristoylated dynamin inhibitory peptide was purchased from Tocris.

MAb 41D3, directed against pSn [5], [9], isotype matched (IgG_1_) control mAb 13D12, directed against pseudorabies virus glycoprotein gD [63] and mAb 74-22-15, reactive with SWC3 on porcine monocytes, macrophages and neutrophils [64] were purified using protein G sepharose column chromatography (Amersham Biosciences), dialyzed to PBS and stored at −70°C. Raft marker GM1 was visualized via cholera toxin B subunit conjugated to HRP or biotin for Western blot analysis and confocal microscopical analysis (Invitrogen – Molecular Probes) respectively. Mouse anti-clathrin IgM antibodies (ICN Biomedicals) and mouse anti-dynein IgM antibodies (Sigma) were used to label clathrin and dynein, respectively. Endosomal markers early endosome antigen 1 (EEA1), cation-independent mannose-6-phosphate receptor (CI-M6P) and lysosome-associated membrane protein 1 (Lamp1) were visualized via an affinity purified goat pAb (sc-6414; Santa Cruz Biotechnology), a rabbit pAb (ab32815; Abcam) and a rabbit pAb (sc-5570; Santa Cruz Biotechnology) respectively. Finally, human serum albumin (HSA) was visualized using biotinylated HSA-specific pig serum [65].

### Pepsinolysis of mouse IgG_1_ antibodies to F(ab')_2_ fragments

F(ab')_2_ fragments were generated essentially as described by Wilson et al. [66]. 300 µg antibodies in 300 µl PBS supplemented with G7 reaction buffer were treated with 10 units/µl PNGase F (New England Biolabs) for 6 hrs to deglycosylate the antibodies. The subsequent pepsinolysis reaction consisted of 30% by volume pepsin agarose beads (Thermo scientific – Pierce) (washed in 20 mM NaOAc, pH 4.5), 20% by volume 5x pepsinolysis buffer (163 mM NaOAc, 1 M KCl, 0.5% Triton X-100, pH 3.5), and 50% by volume deglycosylation reaction. The pepsin treatment was carried out for 16 hrs. Finally, Fc fragments and buffer components were removed by extensive dialysis against PBS using a slide-a-lyzer cassette (Thermo scientific – Pierce) with a 10 kDa cut off. F(ab')_2_ fragments were analyzed by SDS-PAGE and Western blotting to ensure complete removal of the Fc domain. Detection was performed using HRP-labelled goat anti-mouse IgG (Fc specific) antibodies (Sigma Aldrich) and HRP-labelled goat anti-mouse IgG antibodies (Dako).

### Construction of lentiviruses and lentiviral transduction

The lentiviral TRIPΔU3-CMV-WPRE vector ( = TRIPΔU3-CMV-GFP-WPRE in which GFP was deleted by BamHI-SalI digestion) was used as transfer vector, pMD.G as envelope plasmid and p8.91 as packaging plasmid as described before [67]. The enhanced green fluorescent protein (EGFP) tagged dominant-negative (DN) Eps15 construct, named DIII, and the EGFP tagged control construct DIIIΔ2 [68] were cloned into the TRIPΔU3-CMV-WPRE vector, and lentiviral supernatant was produced and collected as described [22].

Three hours post seeding, primary porcine alveolar macrophages were washed once and medium was replaced with lentiviral supernatant. Twenty hours later, cells were washed and fresh medium was added. Another forty-eight hours later, endocytosis assays were performed as described below.

### Endocytosis assay

Macrophages were incubated with purified antibodies at a concentration of 25 µg/ml for 1 h at 4°C to allow only attachment, but no internalization. Cells were then washed to remove unbound antibody and shifted to 37°C to start endocytosis. After different times, cells were fixed with 3% paraformaldehyde (PF), permeabilized with 0.1% Triton X-100, and stained with FITC-labelled goat-anti-mouse IgG to visualize antibodies bound to and internalized in the cells. As a control, cells were fixed after the 4°C incubation (time 0). To analyze the effect of different drugs on endocytosis, cells were incubated with the indicated concentrations of the drugs before and during the internalization assay, and fixed at 60 min post internalization. Prior to administration, the inhibitors were diluted in their solvent to obtain the different concentrations. Subsequently cells were treated with the same volume but different concentrations of the inhibitors. Treatment without inhibitor was used as negative control. After fixation, Sn was stained and the number of vesicles internalized in the macrophages was counted using confocal microscopy. The effectiveness and specificity of the endocytosis inhibitors was confirmed with known controls, more specifically biotinylated transferrin for clathrin-mediated endocytosis, FITC-labelled BSA for lipid raft/caveolae-mediated endocytosis and fluorescent polystyrene beads with a diameter of 1 µm for phagocytosis [22].

### Analysis of Sn endocytosis

Macrophages were incubated for 1 h at 4°C with mAb 41D3 or irrelevant mAb 13D12, washed to remove unbound antibody, and shifted to 37°C. At different time points, cells were cooled to 4°C to stop the endocytosis process, fixed, and stained with FITC-labelled goat-anti-mouse IgG to detect only surface bound 41D3, but not internalized antibody.

### Flow cytometric analysis of pSn endocytosis

Sn endocytosis was quantified by flow cytometry as described [49]. Macrophages were chilled on ice for 30 min, then incubated with 10 µg/ml mAb 41D3 in medium for 1 h at 4°C, and washed to remove unbound mAb. Cells were then shifted to 37°C by the addition of warm medium and further incubated at 37°C. At different time points, endocytosis was stopped by shifting the cells to 4°C. Cells were lifted by incubation with 10 mM EDTA at 4°C, and pelleted by centrifugation (250×g, 5 min, 4°C). Cells were incubated with FITC-labelled goat-anti-mouse IgG for 1 h at 4°C, washed with ice-cold PBS, and analyzed by flow cytometry with a FACScalibur (Becton Dickinson). Forward-scattered light (FSC), sideward-scattered light (SSC) and the FITC fluorescence signal (FL-1) were stored for further analysis.

### Immunofluorescence stainings

For double immunofluorescence stainings, Sn endocytosis was induced using mAb 41D3 as described above and cells were fixed with 3% PF at indicated time points after the 37°C shift.

For clathrin and dynein staining, the cells were washed twice in TBS-GS (50 mM Tris-HCl pH 7.5, 150 mM NaCl, 4.5% sucrose, 2% heat-inactivated goat serum), permeabilized by incubating the cells in 100% methanol for 30 sec at −20°C, and washed twice with TBS-GS. For double staining of Sn and clathrin, the cells were then incubated with FITC-labelled goat-anti-mouse IgG (Molecular Probes) for 1 h at 37°C to visualize Sn, followed by incubation with anti-clathrin IgM antibodies for 1 h at 37°C, biotinylated goat-anti-mouse IgM antibodies for 1 h at 37°C, and Texas Red-labelled streptavidin (Molecular Probes) for 1 h at 37°C. For double staining of Sn and dynein, the cells were incubated with Texas Red-labelled goat-anti-mouse IgG (Molecular Probes) for 1 h at 37°C to visualize Sn, followed by incubation with anti-dynein IgM antibodies for 1 h at 37°C, biotinylated goat-anti-mouse IgM antibodies for 1 h at 37°C, and FITC-labelled streptavidin (Molecular Probes) for 1 h at 37°C.

For double staining of Sn and markers of endosomal compartments, subsequent to 41D3 internalization, cells were fixed, permeabilized and stained for Sn using FITC-labelled goat-anti-mouse IgG. Different compartments of the endocytic pathway were visualized via their respective primary antibodies followed by appropriate TexasRed labelled secondary antibodies.

For double stainings of Sn and lipid raft microdomains on the plasma membrane of macrophages, cells were fixed in 3% PF and incubated for 1 h with mAb 41D3 and 1 h with FITC-labelled goat-anti-mouse IgG (Molecular Probes), followed by incubation for 1 h with biotinylated Cholera toxin B subunit and 1 h with Texas Red-labelled streptavidin.

Detection of cell death upon incubation of macrophages with immunotoxins was done by incubating living cells with ethidium monoazide bromide (EMA) as described [69].

Cells were washed 3 times following each incubation step. At the end, cells were mounted in a glycerin-PBS solution (0.9/0.1, vol/vol) with 2.5% 1,4-diazabicyclo(2.2.2)octane (DABCO; Janssen Chimica).

### Purification of lipid rafts by density ultracentrifugation

Lipid rafts were purified essentially as described previously [70], [71]. Briefly, primary alveolar macrophages were lysed for 30 min at 4°C in TNE buffer (25 mM Tris–HCl, 150 mM NaCl, 5 mM EDTA) containing 1% Triton X-100 and complete protease inhibitor cocktail (Roche). After homogenization with a 25-gauge needle on a 1 ml syringe, the lysate was mixed with ice-cold iodixanol (Optiprep; Nycomed Pharma) up to 40% iodixanol. This mixture was put at the bottom of a Beckman SW41Ti ultracentrifuge tube, overlaid with 5 ml 30% iodixanol (ice-cold) and 3 ml 5% iodixanol (ice-cold), and centrifuged at 200,000×g at 4°C for 20 h in the SW41Ti rotor of a Beckman ultracentrifuge. Ten to twelve fractions were collected from top to bottom, diluted 1∶2 in 2× concentrated non-reducing SDS–PAGE loading buffer and subjected to SDS–PAGE and Western blot. Blots were incubated with the appropriate antibodies or conjugates for detection of Sn, transferrin receptor and GM1, and developed with ECL.

### Confocal laser scanning microscopy

Z-section images of samples were acquired using a Leica TCS SP2 laser scanning spectral confocal system (Leica Microsystems GmbH) linked to a Leica DM IRBE inverted microscope (Leica Microsystems GmbH). Image acquisition was done using the Leica TCS SP2 confocal software package, overlay images were produced with Adobe Photoshop CS and analysis of co-localization was done using CoLocalizer Pro. For the co-localization analysis between Sn and the endosomal markers, the Sn-positive plasma membrane was excluded from the analysis and only internalized Sn was considered.

### Preparation of immunotoxins and HSA immunoconjugates

Purified mAb 41D3 or isotype matched (IgG_1_) control mAb 13D12 were coupled to saporin (Sigma) via a disulfide linker [28]. Both antibody and saporin were labelled with the cross-linker SPDP (N-succinimidyl-3-(2-pyridyldithio)-propionate) according to the manufacturer's instructions (Pierce Biotechnology). The saporin-SPDP was activated via DTT (dithiothreitol, Sigma) and the proteins were purified from the unreacted cross-linkers with PD-10 desalting columns (Amersham Biosciences). Activated proteins were mixed in a 1∶1 antibody∶saporin ratio and incubated for 2 hrs at room temperature. Finally, unreacted saporin was removed from the conjugates by extensive dialysis against PBS using a float-a-lyzer (Spectra/Por) with a 100 kDa cut off. Fractions of all steps were analyzed by SDS-PAGE and Coomassie blue staining to ensure efficient coupling of the products and removal of free saporin.

Human serum albumin (HSA) was coupled to the monoclonal antibodies using a two-step cross-linking protocol. The amine-reactive cross-linker LC-SMCC (Pierce Biotechnology) was coupled to the purified mAb 41D3 or isotype matched (IgG_1_) 13D12 and the amine-reactive cross-linker SPDP (Pierce Biotechnology) was coupled to purified HSA (Sigma) following the manufacturer's instructions. The SPDP-HSA was activated by addition of DTT, which results in the formation of a thiol-activated protein. Both the mAb-LC-SMCC and the thiol-activated HSA were then dialyzed to PBS at 4°C using a membrane with a 10–14 kDa cut-off to remove residual unreacted LC-SMCC, SPDP and DTT. The mAb-LC-SMCC and the thiol-activated HSA were then mixed and incubated at 37°C for 30 min to allow the thiol group on HSA to react with the maleimide end of the LC-SMCC on the mAb, resulting in the formation of a covalent thio-ether bond. To terminate the reaction and to remove unreacted HSA, the mixture was dialyzed against PBS using a membrane with a 100 kDa cut off. Finally, obtained solutions of immunoconjugates were shown negative for LPS content using the Limulus Amebocyte Lysate PYROGENT® Plus assay (Cambrex Bio Science). Samples were taken in between different steps of the cross-linking protocol and analyzed by SDS-PAGE and Coomassie blue staining to confirm that the proteins were cross-linked.

To analyze the capacity of the antibody-antigen immunoconjugates to internalize in macrophages, the 41D3-HSA and 13D12-HSA constructs were also incubated for 1 h at 37°C with primary, Sn-expressing macrophages. Cells were then fixed for 10 min at 37°C with 3% PF, washed and permeabilized with 0.1% Triton X-100. Antibodies were stained by incubation with TexasRed labelled goat-anti-mouse (Molecular Probes). HSA was stained by incubation with biotinylated, HSA-specific polyclonal pig antibodies, followed by incubation with FITC-labelled streptavidin (Invitrogen).

### Immunizations with HSA immunoconjugates and analysis of antibody responses

Six-week-old conventional pigs were housed in isolation units with HEPA filtered air following the recommendations of the Ethical and Animal Welfare Committee of the Faculty of Veterinary Medicine, Ghent University. Six pigs were immunized with 1 mg of 41D3-HSA conjugate, three pigs with 1 mg of a control conjugate (13D12-HSA), three pigs with unconjugated 41D3 and HSA, and three pigs with unconjugated HSA alone. For each pig, half of the sample was injected intramuscularly in 1.5 ml PBS and the other half intravenously in 1.5 ml PBS. Serum samples were collected before immunization (day 0) and at days 10, 17, 24, 32 and 38 after immunization and analyzed for the presence of HSA-specific IgM and IgG antibodies by ELISA as described [65], [72].

## Supporting Information

Figure S1 **Quality control of F(ab')_2_ fragments.** SDS-Page and Western blot analysis of pepsinolysis of control antibody 13D12 and Sn-specific antibody 41D3. – untreated; + treated with PNGase F and pepsin. Antibodies or antibody fragments were visualized with either an Fc-specific HRP-labelled secondary antibody or an HRP-labelled secondary antibody recognizing whole IgG molecules.
(TIF)

Figure S2 **Porcine Sn does not localize to lipid raft microdomains.** (A) Sn is localized in discrete patches on the surface of macrophages. Image represents an overlay of z-sections acquired from top to bottom of a macrophage with surface labelled Sn. (B) Analysis of Sn (green) co-localization with raft marker GM1 (red) on the cell surface. Image is a representative z-section of the top of a macrophage which was surface labelled with mAb 41D3 (Sn) and cholera toxin B subunit (GM1). Scale bar: 5 µm (C) Western blot analysis of fractions obtained by lipid raft flotation assay shows that Sn localizes to fractions enriched in transferrin receptor (non-raft fraction) but not to GM1 enriched fractions (raft fraction).
(TIF)

Results S1 **Porcine sialoadhesin (pSn) does not localize to lipid raft microdomains.**
(DOCX)
