# Supplementary figures and images for: Porcine Sialoadhesin (CD169/Siglec-1) Is an Endocytic Receptor that Allows Targeted Delivery of Toxins and Antigens to Macrophages
Source: PLoS One. 2011 Feb 16;6(2):e16827. doi: 10.1371/journal.pone.0016827 (PMC3040196; doi:10.1371/journal.pone.0016827)

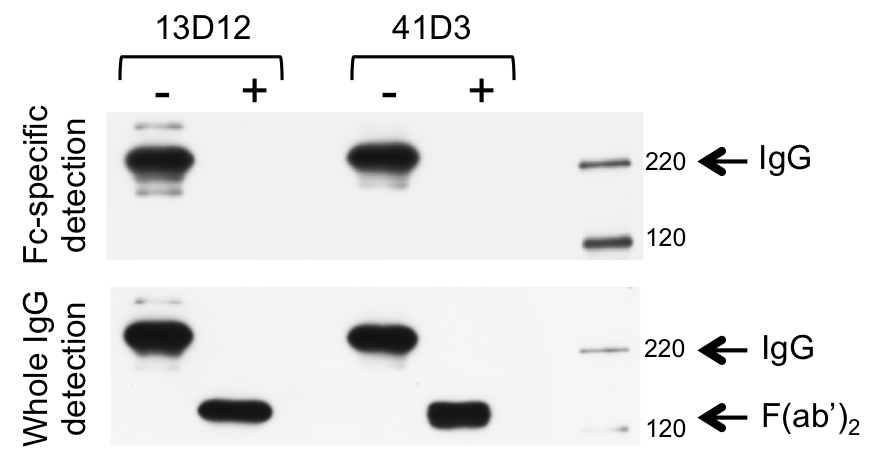

Supplement: Figure S1 — Quality control of F(ab')2 fragments. SDS-Page and Western blot analysis of pepsinolysis of control antibody 13D12 and Sn-specific antibody 41D3. – untreated; + treated with PNGase F and pepsin. Antibodies or antibody fragments were visualized with either an Fc-specific HRP-labelled secondary antibody or an HRP-labelled secondary antibody recognizing whole IgG molecules. (TIF) [file pone.0016827.s001.tif]

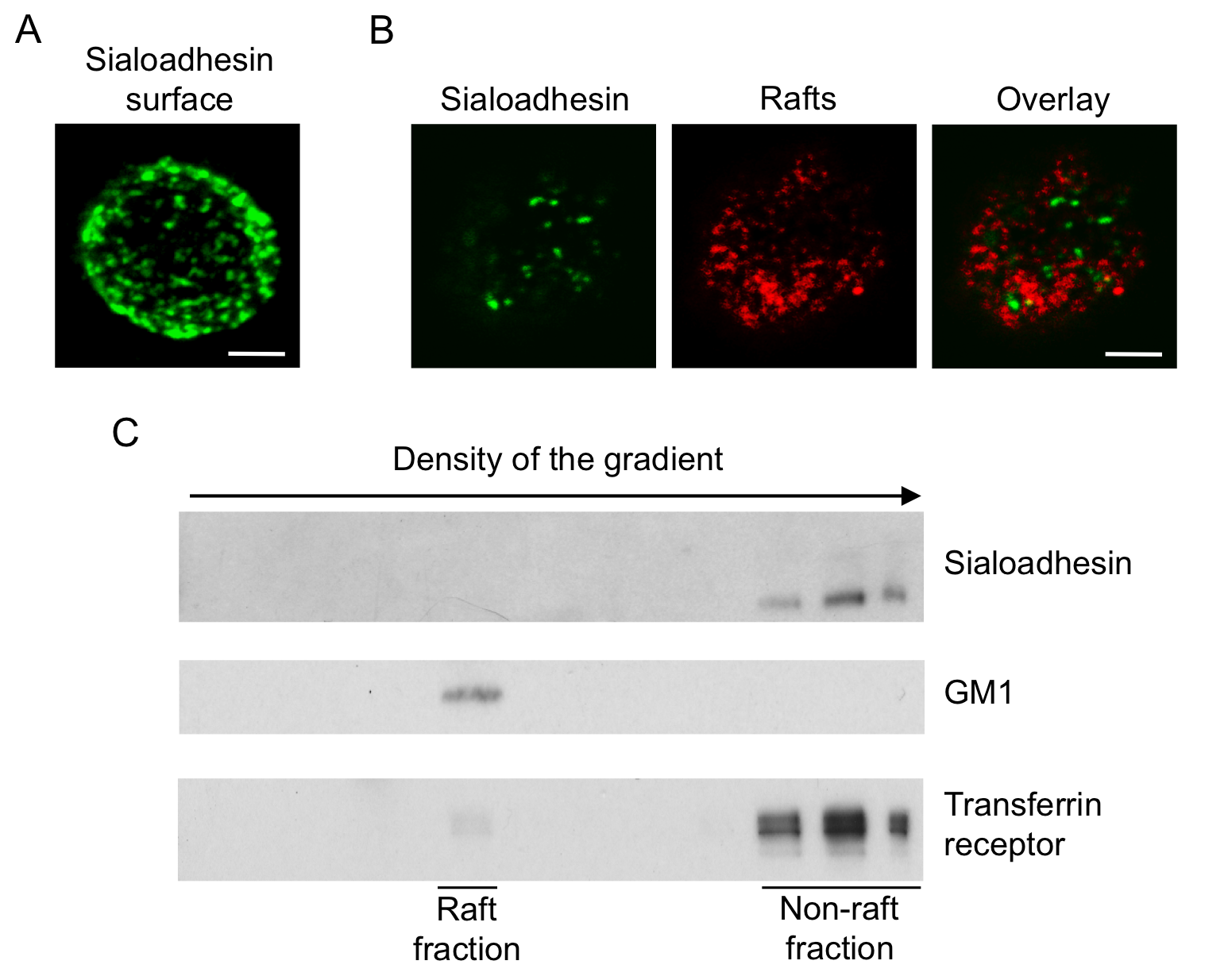

Supplement: Figure S2 — Porcine Sn does not localize to lipid raft microdomains. (A) Sn is localized in discrete patches on the surface of macrophages. Image represents an overlay of z-sections acquired from top to bottom of a macrophage with surface labelled Sn. (B) Analysis of Sn (green) co-localization with raft marker GM1 (red) on the cell surface. Image is a representative z-section of the top of a macrophage which was surface labelled with mAb 41D3 (Sn) and cholera toxin B subunit (GM1). Scale bar: 5 µm (C) Western blot analysis of fractions obtained by lipid raft flotation assay shows that Sn localizes to fractions enriched in transferrin receptor (non-raft fraction) but not to GM1 enriched fractions (raft fraction). (TIF) [file pone.0016827.s002.tif]
