# Supplementary material for: Porcine Sialoadhesin (CD169/Siglec-1) Is an Endocytic Receptor that Allows Targeted Delivery of Toxins and Antigens to Macrophages
Source: PLoS One. 2011 Feb 16;6(2):e16827. doi: 10.1371/journal.pone.0016827 (PMC3040196; doi:10.1371/journal.pone.0016827)
Supplement: Results S1 — Porcine sialoadhesin (pSn) does not localize to lipid raft microdomains. (DOCX) [file pone.0016827.s003.docx]

## Results S1

## Porcine sialoadhesin (pSn) does not localize to lipid raft microdomains

Confocal microscopy reveals an interesting expression pattern of Sn, which can only be detected on the plasma membrane where it is distributed in patches (Fig. S2a) that potentially could be cholesterol enriched lipid raft microdomains. Double immunofluorescence stainings however revealed that pSn present on the plasma membrane does not co-localize with the lipid raft marker GM1 (Fig. S2b). To further investigate the possible localization of Sn in lipid raft fractions, macrophage lipid rafts were isolated using an iodixanol gradient flotation assay [1]. Gradient fractions were analyzed by SDS-PAGE and Western blotting for presence of Sn, raft marker GM1, and the transferrin receptor as non-raft marker. GM1 was present in the upper fractions of the gradient containing the lipid rafts, while transferrin localized essentially to the lower, non-raft fractions, indicating that raft isolation was successful (Fig. S2c). Sn, however, localized only to non-raft fractions, and could not be detected in the raft fractions (Fig. S2c). From these data, it can be concluded that pSn appears not to be localized in GM1 enriched, detergent insoluble microdomains. This is in agreement with the results obtained with the inhibitor nystatin, which interferes with lipid raft/caveolae-mediated endocytosis and had no effect on Sn internalization. Identification and characterization of the Sn-positive microdomains needs further investigation.

1. Favoreel HW, Mettenleiter TC, Nauwynck HJ (2004) Copatching and lipid raft association of different viral glycoproteins expressed on the surfaces of pseudorabies virus-infected cells. J Virol 78: 5279-5287.
